# Prevalence and location of coronary artery disease in anomalous aortic origin of coronary arteries

**DOI:** 10.1097/MCA.0000000000001385

**Published:** 2024-05-15

**Authors:** Sandra Zendjebil, Athanasios Koutsoukis, Thomas Rodier, Fabien Hyafil, Xavier Halna du Fretay, Patrick Dupouy, Jean-Michel Juliard, Reza Farnoud, Phalla Ou, Jean-Pierre Laissy, Camille Couffignal, Pierre Aubry

**Affiliations:** a Department of Cardiology, Assistance Publique-Hôpitaux de Paris, Hôpital Bichat, Paris; b Department of interventional cardiology, Pôle Cardiovasculaire Interventionnel, Clinique les Fontaines, Melun; c Department of Epidemiology, Biostatistics and Clinical Research, Assistance Publique-Hôpitaux de Paris, Hôpital Bichat; d Department of Nuclear Medicine, Assistance Publique-Hôpitaux de Paris, DMU IMAGINA, Hôpital Européen Georges Pompidou, University Paris Cité, Paris; e Department of Cardiology, Pôle Santé Oreliance, Saran; f Department of Radiology, Assistance Publique-Hôpitaux de Paris, Hôpital Bichat, Paris; g Department of Radiology, Centre Hospitalier de Gonesse, Gonesse; h University Paris Diderot, Sorbonne Paris Cité, IAME, INSERM, Paris; i Department of Cardiology, Centre Hospitalier de Gonesse, Gonesse, France

**Keywords:** anomalous aortic origin, coronary artery anomaly, coronary artery disease, coronary computed tomography angiography, invasive coronary angiography

## Abstract

**Background:**

The prevalence and location of coronary artery disease (CAD) in anomalous aortic origin of a coronary artery (AAOCA) remain poorly documented in adults. We sought to assess the presence of CAD in proximal (or ectopic) and distal (or nonectopic) segments of AAOCA. We hypothesized that the representation of CAD may differ among the different courses of AAOCA.

**Methods:**

The presence of CAD was analyzed on coronary angiography and/or coronary computed tomography angiography in 390 patients (median age 64 years; 73% male) with AAOCA included in the anomalous coronary arteries multicentric registry.

**Results:**

AAOCA mainly involved circumflex artery (54.4%) and right coronary artery (RCA) (31.3%). All circumflex arteries had a retroaortic course; RCA mostly an interarterial course (98.4%). No CAD was found in the proximal segment of interarterial AAOCA, whereas 43.8% of retroaortic AAOCA, 28% of prepulmonic AAOCA and 20.8% subpulmonic AAOCA had CAD in their proximal segments (*P* < 0.001). CAD was more prevalent in proximal than in distal segments of retroaortic AAOCA (OR: 3.1, 95% CI: 1.8–5.4, *P* < 0.001). On multivariate analysis, a retroaortic course was associated with an increased prevalence of CAD in the proximal segment (adjusted OR 3.4, 95% CI: 1.3–10.7, *P* = 0.022).

**Conclusion:**

Increased prevalence of CAD was found in the proximal segment of retroaortic AAOCA compared to the proximal segments of other AAOCA, whereas no CAD was observed in the proximal segment of interarterial AAOCA. The mechanisms underlying these differences are not yet clearly identified.

## Introduction

Anomalous aortic origin of coronary arteries (AAOCA) is one of the most frequent congenital cardiac malformation with a coronary computed tomography angiography (CCTA) incidence of 0.8% [1]. Ischemic symptoms related to AAOCA are possible, but they may be also caused by the presence of a coronary artery disease (CAD) in adults. The prevalence of CAD in AAOCA is still poorly documented and conflicting results have been described in the literature [2–10]. Increased prevalence of CAD in circumflex arteries with retroaortic course has been suggested, but sample sizes were often limited and exact location of CAD was not always clearly specified [11–18]. Furthermore, intravascular ultrasonography series suggest that the interarterial course of AAOCA is generally free of CAD [19–24]. Whether some AAOCA predispose to an early CAD or conversely may protect against atherosclerosis development remains unclear. Our objective was to compare the prevalence of CAD in the proximal (or ectopic) and distal (or nonectopic) segments of AAOCA among a large cohort of AAOCA.

## Methods

### Study population

We performed a cross-sectional study from the French noninterventional prospective multicenter ANOCOR (ANOmalous CORonary arteries) registry completed in 2018. Details of the study protocol, recruitment and population have been published elsewhere [25]. The study protocol was conducted with the ethical principles of the 1975 Declaration of Helsinki. This study received ethics committee approval (number IRB00006477) and all patients have given an informed consent for the use of their imaging data for research purpose. Among 472 patients included between January 2010 and January 2013, 496 AAOCA were identified. Twenty-one patients presented more than one AAOCA. Patients (*n* = 16) with nonoptimal image quality of the whole coronary network were excluded. Furthermore, patients (*n* = 63) with the following ANOCOR were excluded: single coronary artery defined as a single ostium with a retrograde filling of the artery not connected with the aorta, anomalous origin within appropriate sinus, high takeoff from ascending aorta, anomalous origin within pulmonary artery, multiple AAOCA, and isolated septal artery AAOCA. Finally 390 patients were included in the study.

### Angiographic analysis

Angiographic images were analyzed by an angiographic committee (cardiologists and radiologists) with expertise in AAOCA. Analysis was performed using invasive coronary angiography (ICA) or CCTA. Each AAOCA was classified following a three-step approach: (a) define the artery presenting the anomalous origin: left main, left anterior descending (LAD) artery, circumflex artery, or right coronary artery (RCA); (b) locate the initial ectopic course in relation with the great vessels: prepulmonic, subpulmonic, interarterial, or retroaortic course; and (c) identify CAD in AAOCA and normal-origin arteries. CAD was evaluated by visual analysis and classified as absent or present (≥1% diameter stenosis). Therefore, nonobstructive CAD (1–49% diameter stenosis) and obstructive CAD (50–100% diameter stenosis) were treated the same way in the final analysis to assess the presence of CAD in each patient. The coronary network (vessels ≥1.5 mm in diameter) was divided into three segments: (a) the proximal (or ectopic) segment of AAOCA, defined as the part from the ostium to the point where the artery reaches its normal course; (b) the distal (or nonectopic) segment of AAOCA, defined as the part of the artery beyond its ectopic course; and (c) the segments of normal-origin arteries, defined as arteries with appropriate aortic connection. The difference between atherosclerotic and nonatherosclerotic diameter narrowing on the proximal segment of AAOCA was based on angiographic characteristics (presence or not of lumen irregularities, plaques, or calcifications) on ICA or CCTA. A proximal narrowing observed in AAOCA with interarterial course was not attributed to CAD it was isolated without any sign of manifest atherosclerosis on coronary imaging.

### Data collection

The set of data included demographic characteristics, cardiovascular risk factors, circumstances of AAOCA diagnosis, and angiographic assessment of all coronary arteries, including the type of AAOCA artery, type of proximal course, and presence of CAD in proximal and distal segments of each AAOCA, and in normal-origin arteries.

### Endpoints

The primary endpoint was CAD prevalence in proximal segment of each AAOCA according to the type of ectopic course (prepulmonic, subpulmonic, interarterial, or retroaortic). The secondary endpoints were CAD prevalence in distal segment of each AAOCA according to the type of ectopic course, the comparison of CAD prevalence between proximal segment and distal segment of each AAOCA, and the comparison of CAD prevalence between each AAOCA with normal-origin arteries

### Statistical analysis

For quantitative variables, descriptive statistics used the median and interquartile range. The discrete variables are presented as number and percentages. Kruskal–Wallis test and Fisher’s exact test were applied where appropriate. A paired univariate logistic regression was performed to estimate the association of CAD in the proximal and distal courses for each type of course. Odds ratio (OR) were reported with two-sided 95% confidence interval (CI) corresponding to the 5% two-tailed test. Univariate and multivariate binary logistic regression were performed on the presence of CAD in the ectopic course of AAOCA. Any variable with *P* < 0.10 in the univariate analysis was included in the model building of multivariable logistic regression, and then a backward elimination strategy was used to delete variables that contribute the least until the final model. We assessed collinearity with Pearson correlation coefficient and VIF (variance inflation factor) method. In the case of Pearson correlation coefficient > 0.8 or VIF > 2.5 between two variables, a collinearity will be approved. Hosmer–Lemeshow test and C-statistic were performed in the final multivariate logistic regression model. All statistical analyses were performed by a dedicated statistician using R software (R Foundation for Statistical Computing, Vienna, Austria. http://www.r-project.org/) v. 4.0. A *P*-value <0.05 was considered to be statistically significant.

## Results

Three hundred and ninety (83%) patients from the ANOCOR registry were included in this study. Angiographic data of the 390 AAOCA were analyzed with ICA, CCTA, or both in 289 (74.1%), 38 (9.7%), and 63 (16.2%) patients, respectively.

### Patients characteristics

Median age was 64 years and 73.1% were male. There was a high prevalence of cardiovascular risk factors including former/current smoker (54.9%), hypertension (54.5%), and dyslipidemia/statin (45.7%). Initial clinical presentation was an acute coronary syndrome in 39.1% of patients and sudden cardiac arrest in four patients (1.2%).

### Anomalous aortic origin of a coronary artery angiographic features

**Fig. 1:**
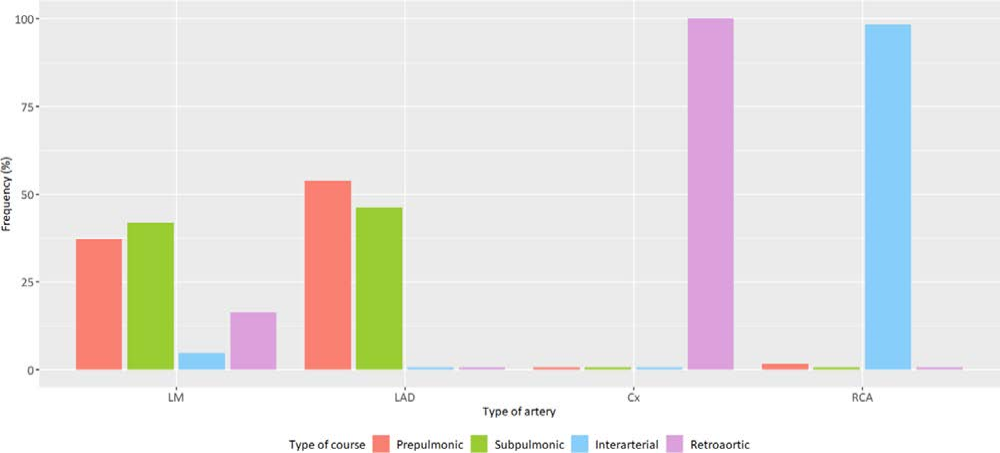
Type of ectopic course according to AAOCA arteries (*n* = 390). AAOCA, anomalous aortic origin of a coronary artery; Cx, circumflex; LAD, left anterior descending; LM, left main; RCA, right coronary artery.

More than half of AAOCA were circumflex arteries (54.4%), followed by RCA (31.3%), left main (11%), and LAD arteries (3.3%). The vast majority (98.4%) of RCA had an interarterial course and all circumflex arteries had a retroaortic course, while several ectopic courses were observed for left main and LAD arteries (Fig. 1). All descriptive baseline data and AAOCA characteristics are presented in Table 1.

**Table 1:** Baseline data and AAOCA characteristics of the study cohort

	*N*	All	Prepulmonic	Subpulmonic	Interarterial	Retroaortic	
		*N* = 390	*N* = 25	*N* = 24	*N* = 122	*N* = 219	*P*-value^a^
Demographics							
Age (years)	390	64.0 (55.0–74.0)	71.0 (64.0–78.0)	67.5 (61.0–79.2)	63.0 (53.2–72.0)	64.0 (55.5–75.0)	0.045
Male gender	390	285 (73.1)	18 (72.0)	18 (75.0)	86 (70.5)	163 (74.4)	0.878
Cardiovascular risk factors							
BMI (kg/m^2^)	369	26.4 (23.9–30.3)	29.4 (25.0–32.8)	25.2 (23.1–29.2)	6.0 (23.6–29.2)	26.8 (24.0–30.4)	0.119
Former/current smoker	386	212 (54.9)	12 (48.0)	9 (40.9)	68 (56.7)	123 (56.2)	0.473
Hypertension	387	211 (54.5)	19 (76.0)	9 (39.1)	65 (53.7)	118 (54.1)	0.075
Dyslipidemia/statin	383	175 (45.7)	14 (56.0)	8 (34.8)	57 (47.9)	96 (44.4)	0.468
Diabetes	381	75 (19.7)	6 (25.0)	6 (26.1)	18 (15.0)	45 (21.0)	0.351
Heredity	382	62 (16.2)	4 (16.7)	5 (21.7)	15 (12.5)	38 (17.7)	0.513
Initial presentation							
No symptom	378	53 (14.0)	1 (4.0)	3 (12.5)	27 (22.7)	22 (10.5)	0.010
Silent ischemia	390	32 (8.2)	1 (4.0)	0 (0.0)	16 (13.1)	15 (6.8)	<0.001
Atypical chest pain	348	39 (11.2)	9 (37.5)	2 (9.1)	10 (9.3)	18 (9.3)	0.003
Stable angina	350	66 (18.9)	3 (13.0)	3 (13.6)	24 (21.4)	36 (18.7)	0.787
NSTEMI	356	90 (25.3)	5 (20.8)	5 (22.7)	25 (22.5)	55 (27.6)	0.715
STEMI	348	48 (13.8)	2 (8.7)	4 (18.2)	15 (13.6)	27 (14.0)	0.832
Dyspnea	360	116 (32.2)	11 (45.8)	9 (37.5)	28 (25.2)	68 (33.8)	0.167
Palpitations	346	37 (10.7)	4 (17.4)	2 (9.1)	10 (9.2)	21 (10.9)	0.649
Dizziness	346	30 (8.7)	2 (8.7)	0 (0.0)	9 (8.3)	19 (9.9)	0.569
Syncope	346	8 (2.3)	1 (4.4)	0 (0.0)	2 (1.8)	5 (2.6)	0.762
Sudden cardiac arrest	345	4 (1.2)	0 (0.0)	0 (0.0)	0 (0.0)	4 (2.1)	0.601

AAOCA	390						–
Left main		43 (11.0)	16 (64.0)	18 (75.0)	2 (1.6)	7 (3.2)	
LAD artery		13 (3.3)	7 (28.0)	6 (25.0)	0 (0.0)	0 (0.0)	
Circumflex artery		212 (54.4)	0 (0.0)	0 (0.0)	0 (0.0)	212 (96.8)	
Right coronary artery		122 (31.3)	2 (8.0)	0 (0.0)	120 (98.4)	0 (0.0)	

Results are expressed as *n* (%) or median (interquartile range).

AAOCA, anomalous aortic origin of a coronary artery; LAD, left anterior descending; NSTEMI, non-ST elevation myocardial infarction; STEMI, ST elevation myocardial infarction.

a Kruskal-Wallis test for continuous variables and Chi-squared test or exact Fisher test for categorical variables.

### Coronary artery disease in anomalous aortic origin of a coronary artery and normal-origin arteries

**Fig. 2:**
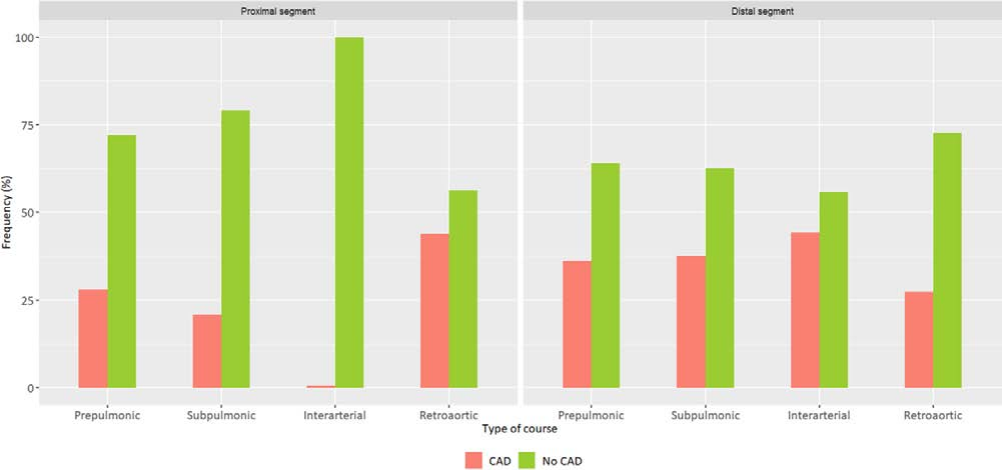
CAD prevalence in the proximal and distal segments according to the type of AAOCA course. AAOCA, anomalous aortic origin of a coronary artery; CAD, coronary artery disease.

Presence of CAD (AAOCA or normal-origin arteries) was observed in 296 patients (75.9%). Analysis of proximal segments of AAOCA showed higher prevalence of CAD in AAOCA with retroaortic course (43.8%) compared to AAOCA with prepulmonic course (28%) or subpulmonic course (20.8%) (*P* < 0.001) (Table 2). Noticeably, no CAD was detected in proximal segments of AAOCA with interarterial course. Analysis of distal segments of AAOCA showed higher prevalence of CAD in AAOCA with interarterial course (44.3%) compared to AAOCA with retroaortic course (27.4%) (*P* = 0. 017). CAD was observed in 36% of AAOCA with a prepulmonic course and 37.5% of AAOCA with a subpulmonic course. All CAD features of AAOCA are presented in Fig. 2. No significant difference was found in the prevalence of CAD in normal-origin arteries according to the type of AAOCA.

**Table 2:** Distribution of CAD in AAOCA and normal-origin arteries

	*N*	AAOCA arteries				Normal-origin arteries	
		Proximal segment		Distal segment			
		CAD	*P*-value^a^	CAD	*P*-value^a^	CAD	*P*-value^a^
		*N* = 108 (27.7%)		*N* = 132 (33.8%)		*N* = 280 (71.8%)	
Type of course	390		<0.001		0.017		0.147
Prepulmonic	25	7 (28.0)		9 (36.0)		21 (84.0)	
Subpulmonic	24	5 (20.8)		9 (37.5)		15 (62.5)	
Interarterial	122	0 (0.0)		54 (44.3)		81 (66.4)	
Retroaortic	219	96 (43.8)		60 (27.4)		163 (74.4)	

Results are expressed as n (%).

AAOCA, anomalous aortic origin of a coronary artery; CAD, coronary artery disease.

a Chi-squared test or exact Fisher test.

### Coronary artery disease prevalence between proximal and distal segments of anomalous aortic origin of a coronary artery

AAOCA with retroaortic course had a higher prevalence of CAD in proximal segment in comparison to distal segment (OR = 3.1; 95% CI: 1.8–5.4; *P* < 0.001) (Table 3). AAOCA with prepulmonic or subpulmonic course were not associated with a higher prevalence of CAD in proximal segment in comparison to distal segment (OR = 0.7; 95% CI: 0.2–2.3; *P* = 0.60 and OR = 0.4; 95% CI: 0.1–1.7; *P* = 0.20, respectively). No OR could be calculated between proximal and distal segments of AAOCA with interarterial course due to the absence of CAD in the proximal segment.

**Table 3:** Comparison of CAD prevalence in proximal and distal segments of AAOCA

Type of course	CAD *N* (%)	Crude OR	95% CI	*P*-value^a^
Prepulmonic (*N* = 25)				
Proximal segment	7 (28.0)	0.7	0.2–2.3	0.566
Distal segment	9 (36.0)			
Subpulmonic (*N* = 24)				
Proximal segment	5 (20.8)	0.4	0.1–1.7	0.220
Distal segment	9 (37.5)			
Interarterial (*N* = 122)				
Proximal segment	0 (0.0)	Not estimated		
Distal segment	54 (44.3)			
Retroaortic (*N* = 219)				
Proximal segment	96 (43.8)	3.1	1.8–5.4	<0.001
Distal segment	60 (27.3)			

Results are expressed as *n* (%).

AAOCA, anomalous aortic origin of a coronary artery; CAD, coronary artery disease; CI, confidence interval; OR, odds ratio.

a Conditional logistic regression paired at the ectopic course binary variable.

### Risk factors for coronary artery disease in proximal segment of anomalous aortic origin of a coronary artery

In multivariate analysis, the presence of a retroaortic course was confirmed as an independent predictor of CAD in the proximal segment of AAOCA (OR = 3.4, 95% CI: 1.3–10.7; *P* = 0.022). Male gender was associated to higher prevalence of CAD in the proximal segment of AAOCA (OR = 2.6; 95% CI: 1.4–5.0; *P* = 0.003) (Table 4). The evaluation of this model resulted in a C-statistic equal to 0.80 (95% CI: 0.76–0.85) and a nonsignificant Hosmer–Lemeshow test *(P* = 0.93).

**Table 4:** Univariate and multivariate logistic regression analysis for CAD in proximal segment of AAOCA

	*N*	Univariate analysis			Multivariate analysis		
		Crude OR	95% CI	*P*-value^a^	Adjusted OR	95% CI	*P*-value^b^
Types of course	390						
Prepulmonic	25	1.50	0.40–5.80	0.561	1.50	0.40–6.10	0.538
Subpulmonic	24	1.00			1.00		
Interarterial	122	Not estimated			Not estimated		
Retroaortic	219	2.97	1.20–9.20	0.037	3.40	1.30–10.70	0.022
Age	390	1.02	1.02–1.04	0.029	1.00	1.01–1.05	0.014
Male gender	390	2.06	1.20–3.70	0.011	2.60	1.40–5.00	0.003
BMI	369	1.02	0.98–1.07	0.292			
Former/current smoker	386	1.55	0.98–2.46	0.061			
Hypertension	387	1.06	0.68–1.66	0.799			
Dyslipidemia/statin	383	1.21	0.77–1.89	0.414			
Diabetes	381	1.19	0.68–2.04	0.540			
Heredity	382	1.52	0.84–2.69	0.154			

AAOCA, anomalous aortic origin of a coronary artery; CAD, coronary artery disease; CI, confidence interval; OR, odds ratio.

a Wald test. Variables with *P*-value <0.1 were integrated in the multivariate analysis.

b Wald test. Multivariate analysis carried out from 360 observations.

### Coronary artery disease association between normal-origin arteries and anomalous aortic origin of a coronary artery

Presence of CAD in normal-origin arteries was associated with higher risk of CAD in the proximal and distal segments of AAOCA for the same patient (OR = 8.4; 95% CI: 4.0–20.6; *P* < 0.001 and OR = 7.7; 4.0–16.4; *P* < 0.001, respectively).

## Discussion

**Fig. 3:**
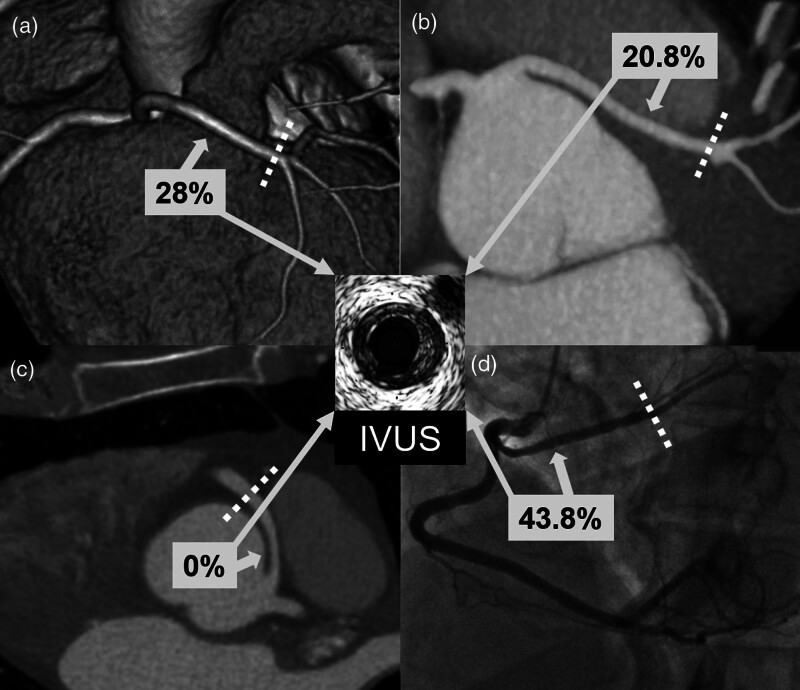
Illustration of CAD prevalence (%) in the proximal segment according to the type of AAOCA course. Panel A: CT image of a left main with a prepulmonic course; panel B: CT image of a left main with a subpulmonic course; panel C: CT image of a right coronary artery with an interarterial course; panel D: angiographic view of a circumflex artery with a retroaortic course. Dotted lines indicate the border between the proximal and distal segments. AAOCA, anomalous aortic origin of a coronary artery; CAD, coronary artery disease; CT, computed tomography; IVUS, intravascular ultrasound.

**Fig. 4:**
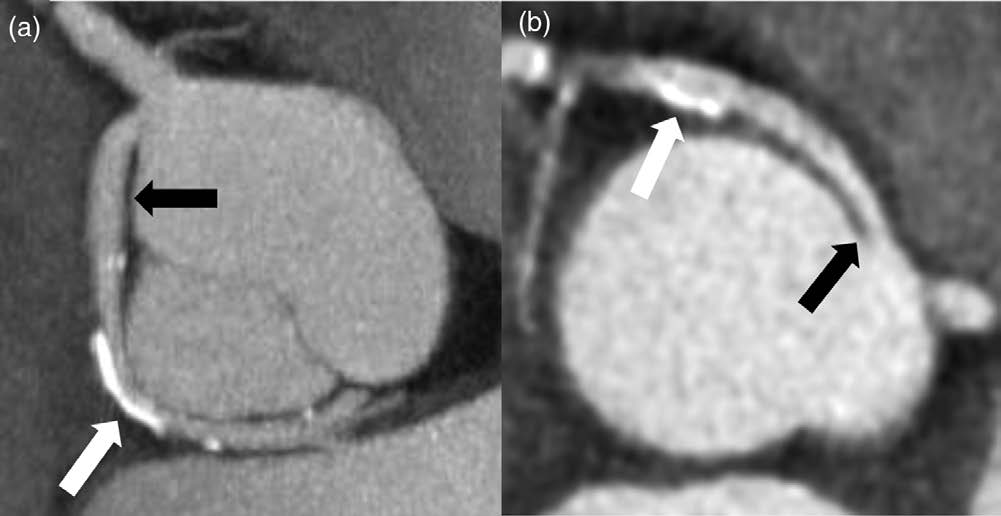
Illustration of CAD presence in different AAOCA. Panel A: CT image of a circumflex artery (black arrow) arising from the right sinus and associated with a retroaortic course. Presence of a calcified plaque (white arrow) on the ectopic segment. Panel B: CT image of a right coronary artery (black arrow) arising from the left sinus and associated with an interarterial course. Presence of a calcified plaque (white arrow) on the nonectopic segment. To note, the ectopic segment is free from CAD. AAOCA, anomalous aortic origin of a coronary artery, CAD, coronary artery disease; CT, computed tomography.

In this study, based on a large AAOCA registry, we found significant differences in the prevalence of CAD between the different ectopic courses of AAOCA (Fig. 3). The presented data showed an increased prevalence of CAD in the proximal segment of AAOCA with retroaortic course, mostly in circumflex arteries (Fig. 4). In addition, we observed the absence of CAD in the proximal segment of AAOCA with interarterial course, mostly in RCA (Fig. 4).

### Coronary artery disease prevalence and location in anomalous aortic origin of a coronary artery

The prevalence of CAD in AAOCA or normal-origin arteries was high (three-quarters of the patients), but adequate with the risk profile of the cohort. To date, it remains unclear in the literature whether the CAD prevalence differs in AAOCA compared to normal-origin arteries, as well as among the AAOCA. Our results showed the increased prevalence of CAD (43.8%) in the proximal segment of AAOCA with retroaortic course in comparison to proximal segments with prepulmonic, subpulmonic, or interarterial course. In addition, we found that the presence of a retroaortic course was an independent predictor of CAD in the proximal segment of AAOCA. These results, based on a large cohort (>200 cases), are consistent with former observations in smaller series (3–38 cases) showing increased prevalence of CAD in the proximal course of circumflex arteries [12,13,15–17]. It appears important to discriminate proximal from distal segments of AAOCA in the analysis of the prevalence of CAD. The interpretation of data without details on the location of CAD must be made with caution [2,6,7,11,14,18]. In a recent study, circumflex artery was associated with the highest prevalence of CAD (54%), but the location of CAD was not specified [18]. In our study, we also confirmed the absence of CAD in the proximal segment of all AAOCA with interarterial course. These results, based on a large cohort (>100 cases) are in line with ones observed in smaller series (3–42 cases) using intravascular ultrasound imaging [19–22,24]. As mentioned above, the results of studies pooling the prevalence of CAD both in proximal course and distal segments of RCA remain less relevant [2,3,5–7,10,18]. In opposite to RCA and circumflex arteries, we found no clear scheme for the prevalence of CAD in left main and LAD arteries. This may be due to the lack of power (few cases for each and different possible courses).

### Mechanisms of coronary artery disease and anomalous aortic origin of a coronary artery

Several mechanisms to explain the higher prevalence of CAD in the proximal segment of AAOCA with retroaortic course have been suggested previously, but none have yet been validated. Changes in the hemodynamics with high flow disturbances leading to greater CAD development appear as a possible mechanism. Retroaortic course is characterized by a long path with a concave marked curve. Other ectopic courses have a convex curve (prepulmonic course and interarterial course), or less marked concave curve (subpulmonic course). Modeling and simulation of fluid systems through the different ectopic courses could be of interest.

The absence of CAD in the proximal segment in AAOCA with interarterial course remains intriguing. Some anatomic characteristics could be evoked, as the lack of perivascular tissues or less, due to the restricted space between the great vessels. Bidirectional communication exists between vascular wall and perivascular adipocytes with a proatherogenic role for the latter [26]. Further studies could investigate the potential differences of perivascular fat presence both in proximal and distal segments of AAOCA. Computed tomography imaging can now evaluate the perivascular fat surrounding the coronary arteries [27]. Of importance, stenosis with variable degree, observed by ICA or CCTA in interarterial course, should not be interpreted as atherosclerotic lesion [28]. An intramural aortic passage is often present in AAOCA with interarterial course. The coronary wall is then embedded into the aortic wall without adventitia between the two walls. Adventitia plays a role in atherosclerotic plaque development through its vasa vasorum. Thus, the low predisposition of interarterial course to CAD could be explained by a different organization of the vascular wall layers and adjacent tissues [29].

The possibility of alteration of the coronary flow downstream of the ectopic course appears unlikely. We, however, found a higher CAD prevalence in the distal segment of AAOCA with interarterial course in comparison with other ectopic courses. There is no clear explication for this unexpected finding. Our observation is in contrast with the results of a large cohort of 128 RCA [8].

### Strengths and limitations

The major strength of this study is the size of the cohort recruited, the largest published, to our knowledge, in adult population. In addition, we chose to compare the prevalence of CAD between the proximal segments of AAOCA drawing a clear distinction between the proximal and distal segments of each AAOCA. This study presents several limitations. First, a propensity score matching with patients free of AAOCA was not made to reduce the bias due to confounding variables. The large sample sizes for AAOCA with interarterial course (>100) or retroaortic course (>200), however, may limit the risk of erroneous explanations of our conclusions. Second, we had relatively few cases of LAD and left main arteries in our AAOCA cohort, which does not allow us to draw any conclusion on these arteries associated with several types of ectopic course. Third, few patients had intravascular ultrasound imaging to formally eliminate the associated presence of CAD in proximal segments of AAOCA. A non-acquired proximal narrowing is frequent in AAOCA with interarterial course because the arterial development must adapt to the limited space between the great vessels. The angiographic analysis, however, was performed by cardiologists and radiologists with expertise in AAOCA. Finally, the severity of CAD was not taken into account in this study. Nevertheless, the objective was to identify CAD even prematurely in AAOCA, and not the association between CAD and symptoms or myocardial ischemia.

### Perspectives

Patients with retroaortic AAOCA have high prevalence of CAD in proximal segments. The question of preventive measures, as the use of statins and optimal control of cardiovascular risk factors, against an early CAD development, might be addressed for this population. In patients with interarterial AAOCA, the proximal segment appears to be protected from CAD. These observations open new insights into the role of perivascular space in the development of CAD.

### Conclusion

The current study, the largest to our knowledge to analyze the prevalence of CAD in AAOCA in adults, confirms that the proximal segment of AAOCA with retroaortic course is particularly exposed to the development of CAD, while the proximal segment of AAOCA with interarterial course seems to be protected from this disease. The mechanisms underlying these differences remain to be identified.

## Acknowledgements

The authors thank all investigators of this study for their contribution to the construction of the ANOCOR cohort.

This work was supported by a grant from the Groupe Athérome et Cardiologie Interventionnelle (GACI) of the French Society of Cardiology. The funder did not have any role in the design of the study, analysis and interpretation of the data, or writing of the results for publication.

S.Z. and P.A. conceived and designed the study. R.F. organized the database and extracted data. T.R. and C.C. performed the statistical analysis and created all tables and figures. S.Z., A.K., F.H., X.H.F., P.D., J.M.J., P.O., J.P.L., and P.A. analyzed and interpreted the results. S.Z. and P.A. wrote the first draft of the manuscript. All authors provided intellectual input, contributed to manuscript revision and approved the final version.

ANOCOR investigators: Wissam Abi Khalil, Luc Aguirre, Abdel Akesbi, Pierre Aubry, Yves Banus, Loic Belle, Hakim Benamer, Yves Biron, Emmanuel Boiffard, Rachid Bouallal, Olivier Boudvillain, Ryad Bourkaïb, Camille Brasselet, Erwan Bressollette, Philippe Brunel, Didier Champagnac, Michel Coco, Philippe Commeau, Stephane Cook, Philippe Couppie, Fabien de Poli, Laurent Delorme, Fleur Descoutures, Romain Didier, Gregory Ducrocq, Patrick Dupouy, Chloé Durier, Rami El Mahmoud, Jean-Baptiste Estève, Benjamin Faurie, Eric Garbarz, Jean-Louis Georges, Benoit Gerardin, Géraldine Gibault-Genty, Martine Gilard, Matthieu Godin, Jean-Jacques Goy, Claire Haffner-Debus, Xavier Halna du Fretay, Michel Hanssen, Sébastien Hascoët, Laurent Jacquemin, Julien Jeanneteau, Thierry Joseph, Jean-Michel Juliard, Bernard Karsenty, René Koning, Eugenio La Scala, Pierre Leddet, Gilles Lemesle, Guillaume Leurent, Raphy Levy, Bernard Livarek, Christophe Loubeyre, Luc Maillard, Lionel Mangin, Stéphanie Marlière, Mohammed Nejjari, Patrick Ohlmann, Nabil Poulos, Antoine Py, Sylvain Ranc, Alain Rialan, Ricardo Roriz, Pierre Rougier, Patrick Staat, Christophe Thuaire, Mario Togni, Jérôme van Rothem, Olivier Varenne, and Vassilis Voudris.

### Conflicts of interest

There are no conflicts of interest.

## References

[1] Cheezum MK Liberthson RR Shah NR Villines TC O’Gara PT Lanszberg MJ. Anomalous aortic origin of a coronary artery from the inappropriate sinus of valsalva. J Am Coll Cardiol 2017; 69:1592–1608. 28335843 10.1016/j.jacc.2017.01.031

[2] Chaitman BR Lespérance J Saltiel J Bourassa MB. Clinical, angiographic, and hemodynamic findings in patients with anomalous origin of the coronary arteries. Circulation 1976; 53:122–131. 1244233 10.1161/01.cir.53.1.122

[3] Topaz O DeMarchena EJ Perin E Sommer LS Mallon SM Chahine RA. Anomalous coronary arteries: angiographic findings in 80 patients. Int J Cardiol 1992; 34:129–138. 1737663 10.1016/0167-5273(92)90148-v

[4] Garg N Tewari S Kapoor A Gupta DK Sinha N. Primary congenital anomalies of the coronary arteries: a coronary arteriographic study. Int J Cardiol 2000; 74:39–46. 10854679 10.1016/s0167-5273(00)00243-6

[5] Jim MH Siu CW Ho HH Miu R Lee SW. Anomalous origin of the right coronary artery from the left coronary sinus is associated with early development of coronary artery disease. J Invasive Cardiol 2004; 16:466–468. 15353826

[6] Zhang F Ge JB Qian JY Fan B Wang QB Chen HZ. Frequency of the anomalous coronary origin in the Chinese population with coronary artery stenosis. Zhonghua Nei Ke Za Zhi 2005; 44:347–349. 16009003

[7] Eid AH Itani Z Al-Tannir M Sayegh S Samaha A. Primary congenital anomalies of the coronary arteries and relation to atherosclerosis: an angiographic study in Lebanon. J Cardiothorac Surg 2009; 4:58. 19874587 10.1186/1749-8090-4-58 PMC2775738

[8] Suryanarayana P Kollampare S Riaz IB Lee J Husnain M Luni FK. Presence of anomalous coronary seen on angiogram is not associated with increased risk of significant coronary artery disease. Int J Angiol 2014; 23:243–246. 25484555 10.1055/s-0034-1384839 PMC4244244

[9] Çanga Y Güvenç TS Karataş MB Çalik AN Onuk T Tanik VO. Congenital coronary artery anomalies in adults: review of 111 cases from a single-center experience. Cardiol Young 2017; 27:1041–1050. 27842619 10.1017/S1047951116001906

[10] Sidhu NS Wander GS Monga A Kaur A. Incidence, characteristics and atherosclerotic involvement of coronary artery anomalies in adult population undergoing catheter coronary angiography. Cardiol Res 2019; 10:358–368. 31803334 10.14740/cr941 PMC6879043

[11] Page HL Jr Engel HJ Campbell WB Thomas CS Jr. Anomalous origin of the left circumflex coronary artery. Recognition, angiographic demonstration and clinical significance. Circulation 1974; 50:768–773. 4417692 10.1161/01.cir.50.4.768

[12] Silverman KJ Bulkley BH Hutchins GM. Anomalous left circumflex coronary artery: ‘normal’ variant of uncertain and pathologic significance. Am J Cardiol 1978; 41:1311–1314. 665538 10.1016/0002-9149(78)90891-3

[13] Wilkins CE Betancourt B Mathur VS Massumi A De Castro CM Garcia E. Coronary artery anomalies: a review of more than 10,000 patients from the Clayton cardiovascular laboratories. Tex Heart Inst J 1988; 15:166–173. 15227247 PMC324820

[14] Click RL Holmes DR Vlietstra RE Kosinski AS Kronmal RA. Anomalous coronary arteries: location, degree of atherosclerosis and effect on survival-A report from the coronary artery surgery study. J Am Coll Cardiol 1989; 13:531–537. 2918156 10.1016/0735-1097(89)90588-3

[15] Leroy F Germain S Bauters C Lablanche JM Bertrand ME. Abnormal origin of the left circumflex artery: clinical, angiographic and prognostic aspect. A propos of 30 cases. Arch Mal Coeur 1992; 85:993–999. 1449347

[16] Samarendra P Kumari S Hafeez M Vasavada BC Sacchi TJ. Anomalous circumflex coronary artery: benign or predisposed to selective atherosclerosis. Angiology 2001; 52:521–526. 11512690 10.1177/000331970105200803

[17] West NEJ McKenna CJ Ormerod O Forfar JC Banning AP Channon KM. Percutaneous coronary intervention with stent deployment in anomalously-arising left circumflex coronary arteries. Catheter Cardiovasc Interv 2006; 68:882–890. 17086531 10.1002/ccd.20807

[18] Jiang MX Brinza EK Ghobrial J Tucker DL Gupta S Rajeswaran J. Coronary artery disease in adults with anomalous aortic origin of a coronary artery. JTCVS Open 2022; 10:205–221. 36004264 10.1016/j.xjon.2022.04.022 PMC9390708

[19] Tsujita K Maehara A Mintz GS Franklin-Bond T Mehran R Stone GW. In vivo intravascular ultrasonic assessment of anomalous right coronary artery arising from left coronary sinus. Am J Cardiol 2009; 103:747–751. 19231346 10.1016/j.amjcard.2008.11.016

[20] de Oliveira DM Gomes V Caramori P. Intravascular ultrasound and pharmacological stress test to evaluate the anomalous origin of the right coronary artery. J Invasive Cardiol 2012; 24:E131–E134. 22684396

[21] Angelini P Uribe C Monge J Tobis JM Elayda MA Willerson JT. Origin of the right coronary artery from the opposite sinus of valsalva in adults: characterization by intravascular ultrasonography at baseline and after stent angioplasty. Catheter Cardiovasc Interv 2015; 86:199–208. 26178792 10.1002/ccd.26069 PMC4657462

[22] Lee SE Yu CW Park K Park KW Suh J-W Cho Y-S. Physiological and clinical relevance of anomalous right coronary artery origination from left sinus of Valsalva in adults. Heart 2016; 102:114–119. 26585987 10.1136/heartjnl-2015-308488

[23] Driesen BW Warmerdam EG Sieswerda G-JT Schoof PH Meijboom FJ Haas F. Anomalous coronary artery originating from the opposite sinus of Valsalva (ACAOS), fractional flow reserve- and intravascular-guided management in adult patients. Catheter Cardiovasc Interv 2018; 92:68–75. 29521471 10.1002/ccd.27578

[24] Aubry P Halna du Fretay X Boudvillain O Degrell P; The ANOCOR Working Group. Place of angioplasty for coronary artery anomalies with interarterial course. Front Cardiovasc Med 2021; 7:596018. 33614737 10.3389/fcvm.2020.596018 PMC7893637

[25] Koutsoukis A Halna du Fretay X Dupouy P Ou P Laissy J-P Juliard J-M. Interobserver variability in the classification of congenital coronary abnormalities: a substudy of the anomalous connections of the coronary arteries registry. Congenit Heart Dis 2017; 12:726–732. 28639359 10.1111/chd.12504

[26] Mancio J Oikonomou EK Antoniades C. Perivascular adipose tissue and coronary atherosclerosis. Heart 2018; 104:1654–1662. 29853488 10.1136/heartjnl-2017-312324

[27] Kotanidis CP Antoniades C. Perivascular fat imaging by computed tomography (CT): a virtual guide. Br J Pharmacol 2021; 178:4270–4290. 34296764 10.1111/bph.15634 PMC8856184

[28] Aubry P Halna du Fretay X Calvert PA Dupouy P Hayfil F Laissy JP. Proximal anomalous connections of coronary arteries in adults. In: Rao PS, editor. Congenital heart disease: selected aspects. Intech; 2012. pp. 83–230.

[29] Tinajero MG Gotlieb AI. Recent developments in vascular adventitial pathobiology: the dynamic adventitia as a complex regulator of vascular disease. Am J Pathol 2020; 190:520–534. 31866347 10.1016/j.ajpath.2019.10.021

